# Impact of image averaging on choroidal quantification using swept-source optical coherence tomography

**DOI:** 10.1186/s12886-025-04189-3

**Published:** 2025-07-01

**Authors:** Xiaotian Wu, Yinglong Lan, Peifeng Zhang, Zhiyang Lin, Yanfeng Jiang, Shenghai Huang, Zi Jin, Yuanyuan Wang, Meixiao Shen, Sisi Chen

**Affiliations:** 1 Eye Hospital and School of Ophthalmology and Optometry, Wenzhou Medical University; Oujiang Laboratory (Zhejiang Lab for Regenerative Medicine, Vision and Brain Health), Wenzhou, Zhejiang, 325000 China; 2 National Clinical Research Center for Ocular Diseases, Eye Hospital, Wenzhou Medical University, Wenzhou, Zhejiang 325027 China

**Keywords:** B-Scan average,, Swept-source optical coherence tomography (SS-OCT), Choroidal vascularity index (CVI), , Choroidal thickness (CT)

## Abstract

**Purpose:**

This study aimed to assess the impact of B-scan averaging on choroidal parameters using swept-source optical coherence tomography (SS-OCT).

**Methods:**

Twenty-two right eyes of healthy adults were scanned using SS-OCT. Each scan included five consecutive 9 mm 18-line radial scans with averaging frames of 4, 8, 16, 32, and 64. Two times of averaging were performed per eye. Choroidal images were analyzed with binarization techniques to calculate choroidal parameters, including luminal volume (LV), stromal volume (SV), choroidal vascularity index (CVI), total choroidal volume (TCV), and choroidal thickness (CT), to compare the changes and stability of these parameters under different B-scans averaging conditions.

**Results:**

CVI showed no significant differences across averaging conditions (*P* > 0.05), while LV, SV, TCV, and CT differed significantly (*P* < 0.05). LV, TCV, and CT showed no significant differences when averaging ≥ 16 frames, whereas SV achieved stability at ≥ 8 frames.

**Conclusion:**

This study demonstrated that SS-OCT imaging of the choroid was unaffected by B-scan averaging in CVI measurements. However, LV, TCV, and CT required averaging ≥ 16 frames, while SV required averaging ≥ 8 frames for consistent results. This finding emphasizes the importance of standardized imaging protocols in clinical practice.

## Background

The choroid, located between the retina and sclera, is rich in blood vessels and provides essential nutrients playing an important role in the maintenance of visual function [1, 2]. The choroid can demonstrate significant structural and functional changes in various chorioretinal diseases, such as pathological myopia [3], age-related macular degeneration [4], diabetic retinopathy [5], central serous chorioretinopathy [6], and glaucoma [7]. Therefore, quantitative analysis of choroid in three-dimensional space is very critical, which not only help in the early diagnosis of the above diseases, but also provide an important guide for clinical treatment [8, 9].

Optical coherence tomography (OCT) is an important tool for fundus imaging to visualize the choroid, which is useful for quantifying choroid accurately [10]. The advances in OCT technology, from spectral-domain OCT (SD-OCT) to swept-source OCT (SS-OCT), have improved the visualization of choroidal imaging through deeper tissue penetration [11, 12]. Moreover, OCT image averaging operation is a simple and useful method to eliminate OCT speckle noise and can further enhance visualization of choroidal signal. Currently, the choroid is quantified by obtaining the choroidal boundaries as well as the internal vascular and stromal portions using image grayscale information. Theoretically, choroidal image quality has a significant impact on its quantification results. Obviously, choroidal image quality is affected by the number of images used in the OCT image averaging. For example, while maximizing the B-scan average frames can effectively reduce speckle noise and enhance choroidal visibility, it may also extend scan durations, leading to increased patient fatigue and potential motion artifacts during image acquisition [13–15]. Researchers hypothesized that different B-scan averaging protocols may introduce uncertainty that affects image quality and the comparability of results across different studies [16, 17]. Therefore, a reasonable selection of the number of B-scan averaging is critical in balancing measurement accuracy and its impact on choroidal three-dimensional parameters assessment. However, there are no studies to analyze in detail the specific effects of image averaging on choroidal quantitative parameters nowadays.

The aim of this study was to evaluate the effects of different B-scan stacking times on the consistency of three-dimensional choroidal parameters such as choroidal thickness (CT), choroidal vascular index (CVI), choroidal stromal volume (SV), and choroidal vascular volume (LV) using SS-OCT. By systematically analyzing the quantitative effects of each B-scan stacking number on these parameters, the study will explore the optimal B-scan stacking number for different choroidal parameters in clinical applications, aiming to provide a reference basis for improving the accuracy of quantitative choroidal measurements and the feasibility of clinical operations.

## Methods

This was a cross-sectional study conducted in the optometry imaging laboratory of Wenzhou Medical University. The study adhered to the tenets of the Declaration of Helsinki and was approved by the Ethics Committee of Wenzhou Medical University (registration number: 2019-078-K-77). Written informed consent was obtained from all subjects prior to the examination.

### Subject

Twenty-two healthy young adults with a mean age of 25.83 ± 1.56 years (13 women and 9 men), a mean refractive error of -3.30 ± 2.28 diopters, and a mean axial length of 25.02 ± 1.22 mm was recruited from the ocular imaging laboratory of Wenzhou Medical University.

An experienced ophthalmologist (SHH) conducted comprehensive examinations, which included automatic refractor (ARK-510, NIDEK, Japan), best-corrected visual acuity (BCVA) assessment, optical biometer (Lenstar 900, Koeniz, Switzerland), and slit-lamp biomicroscopy. All examinations were conducted during regular working hours (9:00 AM to 5:00 PM). Participants with BCVA more than 20/20 and spherical equivalent between − 6.00 D and + 1.00 D were included. The exclusion criteria encompassed the history of ocular surgery and trauma, systemic diseases and relevant ocular treatment. Subjects with conditions that could affect retinal morphology, including hypertension, diabetes, age-related macular degeneration, and other retinopathies, were also excluded. Given that the choroidal structure may vary with smoking [18], coffee consumption [19] and alcohol intake [20], only non-smokers were included in this experiment, and participants refrained from coffee or alcohol intake for at least four hours prior to the experiment. Subjects who were undergoing myopia control treatments such as orthokeratology, multifocal spectacle or contact lens, or atropine therapy were excluded due to the potential effect of these treatments on choroidal thickness [21, 22].

### SS-OCT image acquisition

**Fig. 1:**
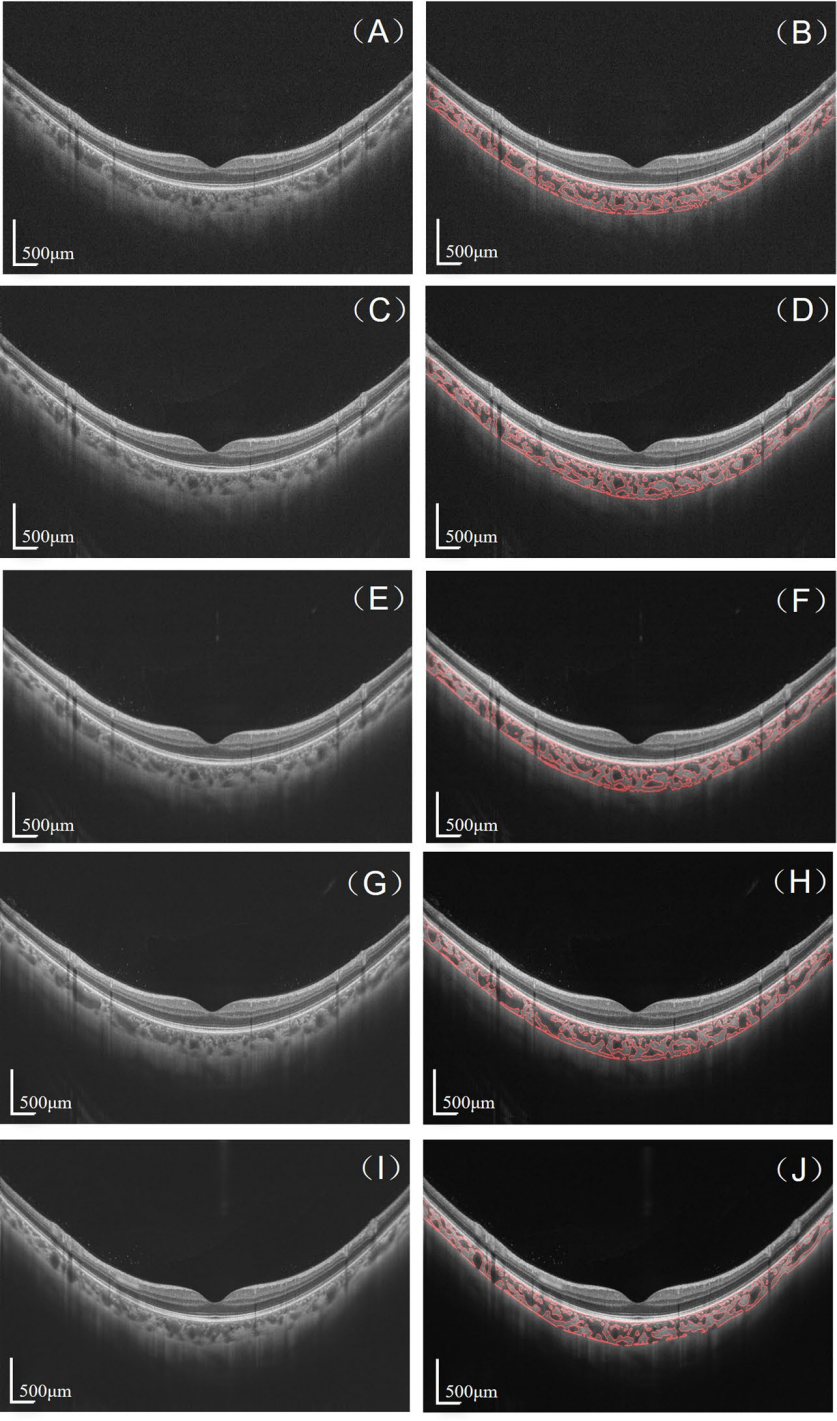
OCT images (**A**, **C**, **E**, **G**, **I**) and binarized choroidal vessels (**B**, **D**, **F**, **H**, **J**) for B-scan averaging: 4 (**A**, **B**), 8 (**C**, **D**), 16 (**E**, **F**), 32 (**G**, **H**), and 64 (**I**, **J**). Scale bars represent 500 μm

The right eyes of all subjects were imaged using an SS-OCT (VG200S, SVision Imaging, Henan, China) with 18-line radial scan patterns. This SS-OCT system captures cross-sectional images of the choroid with digital axial and transverse resolutions of 6.3 and 20 μm, respectively, at a speed of 200,000 A-scans per second. The scan depth is 3 mm in tissue, and scan width is 9 mm. The averaging protocols set five modes, named 4, 8, 16, 32, and 64 B-scan frames for averaging. Subjects were instructed to fixate on an internal target, and underwent SS-OCT imaging using five averaging modes twice. Before each OCT measurement, participants were required to remove their head from the chin and headrest. OCT images with a signal strength of less than 6 were excluded from the study (Fig. 1).

### Choroidal quantification

**Fig. 2:**
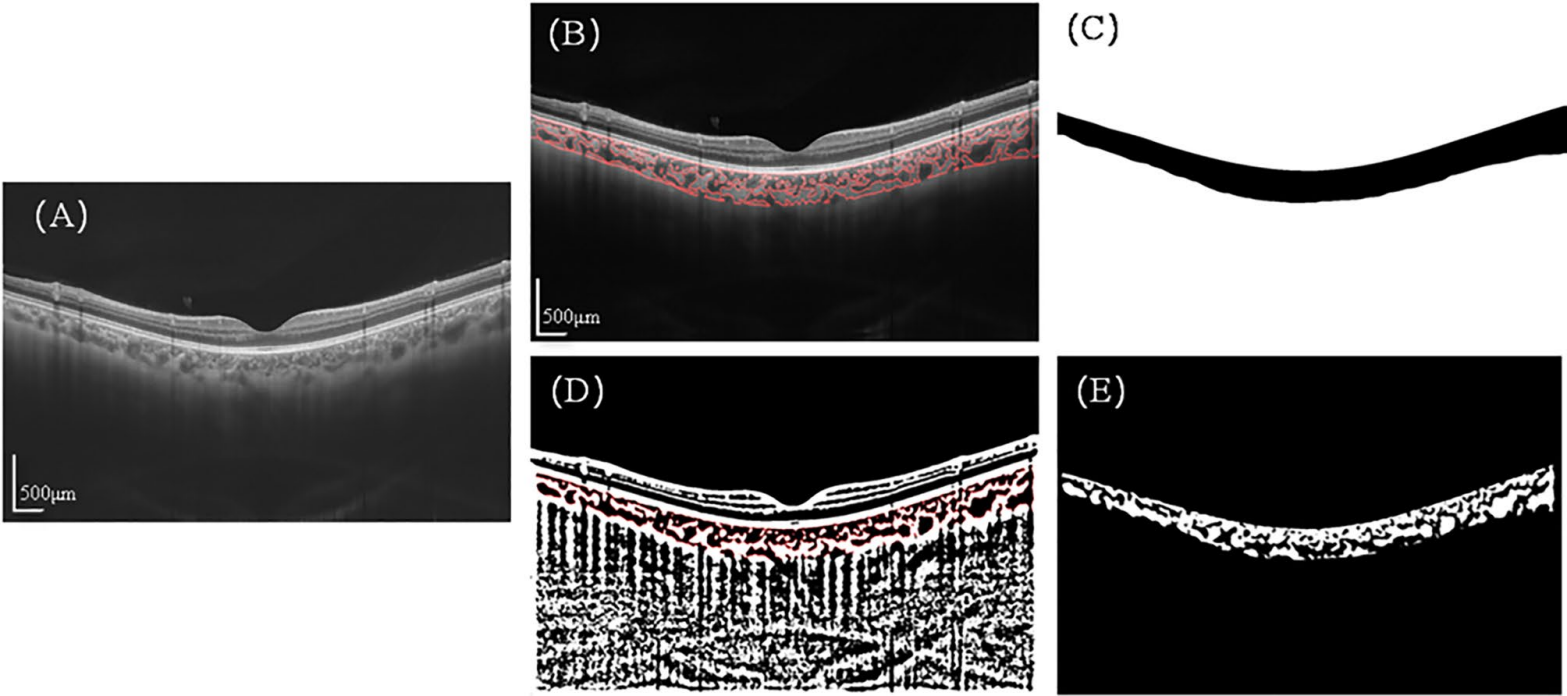
Processing procedure of choroidal images.: (**A**) Original SS-OCT image. (**B**) Overlay of the region of interest created after performing image binarization on the SS-OCT image. (**C**) Segmentation of the choroidal area. (**D**) Binarization image of the SS-OCT image with demarcation of luminal area (LA) and stromal area (SA) using a red dotted line. (**E**) Segmentation of the binarized choroidal region, where white portions represent LA and black portions represent SA

The method for quantifying choroidal parameters, as previously detailed in the literature, entailed two primary steps [23]. The first step was automated segmentation of choroidal boundaries to calculate choroidal thickness (CT). The second step was automated binarization to distinguish between choroidal stroma and lumen, followed by the quantification of the luminal area (LA) and stromal area (SA) in the binarized images. These measurements permit the calculation of the total choroidal area (TCA), and the choroidal vascularity index (CVI) (defined as the ratio of LA to TCA) (Fig. 2). The use of 18 radial and 6 mm range images enables the acquisition of three-dimensional parameters of the choroid. These include luminal volume (LV), stromal volume (SV), total choroidal volume (TCV), and the choroidal vascularity index (CVI), which is defined as the ratio of LV to TCV.

The boundary segmentation algorithm employs gradient information and shortest path search techniques. The upper boundary is identified at the hyper-reflective line corresponding to the RPE-Bruch’s membrane junction, while the lower boundary is determined at the light pixel delineating the choroidal-scleral interface. To enhance accuracy for the challenging choroidal-scleral interface, our algorithm incorporates adaptive contrast enhancement and specialized edge detection filters. This fully automated process demonstrated high reliability across all averaging conditions, ensuring consistent measurement results. The binarization process utilized Niblack’s auto local threshold algorithm implemented in Matlab2017a. In the resulting binary images, LA and SA were represented by values 0 and 1, respectively. The boundary between LA and SA was demarcated for precise quantification. LA was defined as the area of dark pixels, and SA was calculated by subtracting LA from TCA.

### Statistical analysis

Statistical analysis was performed with SPSS 22.0 (SPSS Inc, Chicago, IL, USA). Continuous variables were presented as mean ± standard deviation (SD). To assess intra-observer repeatability of choroidal parameters, two separate sets of images from 22 eyes were collected and measured across five choroidal parameter groups. The coefficient of repeatability (CoR) and intraclass correlation coefficients (ICC), were calculated for each choroidal parameter, under different image averaging conditions, to evaluate measurement consistency. The CoR was expressed as the standard deviation (SD) of the differences between two repeated measurements. Thus, CoR was calculated as $\:1.96*\surd\:2*$ intra-subject standard deviation (S_w_) [24]. Additionally, to assess intra-observer variability, Bland–Altman plots were generated to compare the differences in repeated measurements of each choroidal parameter against their respective mean values. This analysis included calculations of the mean difference, the 95% confidence interval (CI) for the mean difference, and the 95% limits of agreement (LoA), defined as the mean difference ± 1.96 standard deviations (SD).

One-way repeated measures analysis of variance was used to examine the impact of B-scan averaging conditions on choroidal parameters. Initially, One-way analysis of variance (ANOVA) was used to assess the main effects and determine whether the number of B-scan averages significantly influenced the choroidal parameters. Upon identifying significant main effects, pairwise comparisons were conducted using a Bonferroni correction for multiple comparisons. *P*-values less than 0.05 was considered as statistically significant.

## Results

### Repeatability of choroidal quantitative parameters in different averaging modes

**Fig. 3:**
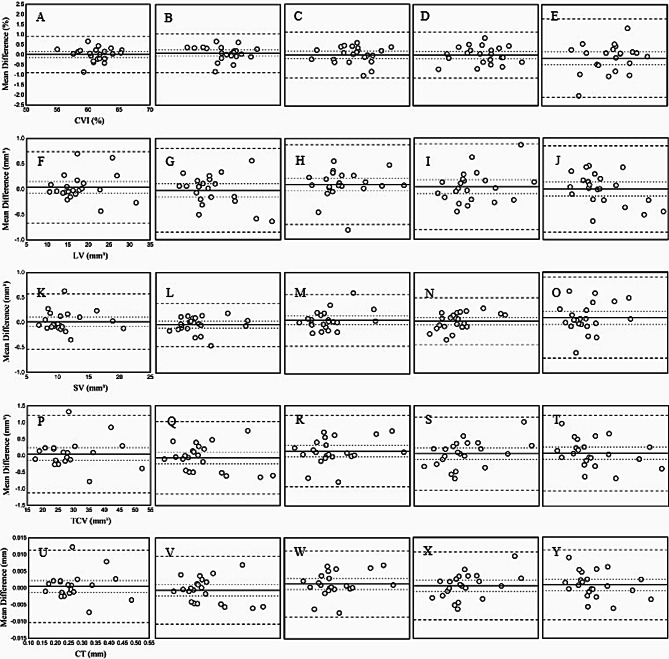
Bland-Altman analysis for repeated measurements of choroidal parameters across all groups: CVI (Choroidal Vascularity Index), LV (Luminal Volume), SV (Stromal Volume), CT (Choroidal Thickness), and TCV (Total Choroidal Volume). (**A**–**E**) CVI at R4, R8, R16, R32, and R64. (**F**–**J**) LV at R4, R8, R16, and R64. (**K**–**O**) SV at R4, R8, R16, R32, and R64. (**P**–**T**) TCV at R4, R8, R16, R32, and R64. (**U**–**Y**) CT at R4, R8, R16, R32, and R64. Here, R4, R8, R16, R32, and R64 indicate an average of 4, 8, 16, 32, and 64 repeated B-scans, respectively. The mean (continuous line), lower and upper limits of agreement [± 1.96 SD (standard deviation), peripheral dotted lines], and the lower and upper confidence intervals (95%) are depicted

Measurements were performed twice for each eye to assess repeatability of choroidal parameters. The ICC for CVI among the five different B-scans averaging groups ranged from 0.977 to 0.991, with CoR ranging from 0.917 to 1.966%. The ICC for LV was consistently 0.999 across all groups, with CoR ranging from 0.714 to 0.864 mm³. For SV, the ICC among the five groups ranged from 0.995 to 0.998, with CoR ranging from 0.435 to 0.815 mm³. Both TCV and CT had an ICC of 0.999 among the five groups, with the CoR of TCV ranging from 1.083 to 1.188 mm³, and the CoR of CT ranging from 0.010 to 0.011 mm(Table 1). The Bland-Altman analysis across five different B-scan averaging conditions demonstrated that the majority of differences between the mean values of the two repeated measurements fell within the limits of agreement (Fig. 3).

**Table 1:** Intraobserver repeatability of choroidal parameters with different numbers of averaged B-scans

	CVI		CT		LV		SV		TCV	
	ICC	CoR(%)	ICC	CoR(mm)	ICC	CoR(mm^3^)	ICC	CoR(mm^3^)	ICC	CoR(mm^3^)
R4	0.991	0.917	0.999	0.011	0.999	0.714	0.998	0.560	0.999	1.188
R8	0.992	0.980	0.999	0.010	0.999	0.841	0.999	0.435	0.999	1.104
R16	0.992	1.141	0.999	0.010	0.999	0.806	0.998	0.519	0.999	1.083
R32	0.993	1.164	0.999	0.010	0.999	0.864	0.998	0.472	0.999	1.105
R64	0.977	1.966	0.999	0.011	0.999	0.860	0.995	0.815	0.999	1.141

ICC: intraclass correlation coefficient, CoR: Coefficient of repeatability, CVI: choroidal vascularity index, CSI: choroidal stromal index, LV: luminal volume, SV: stromal volume, TCV: total choroidal volume, CT: choroidal thickness

### Difference of choroidal quantitative parameters among various averaging conditions

**Fig. 4:**
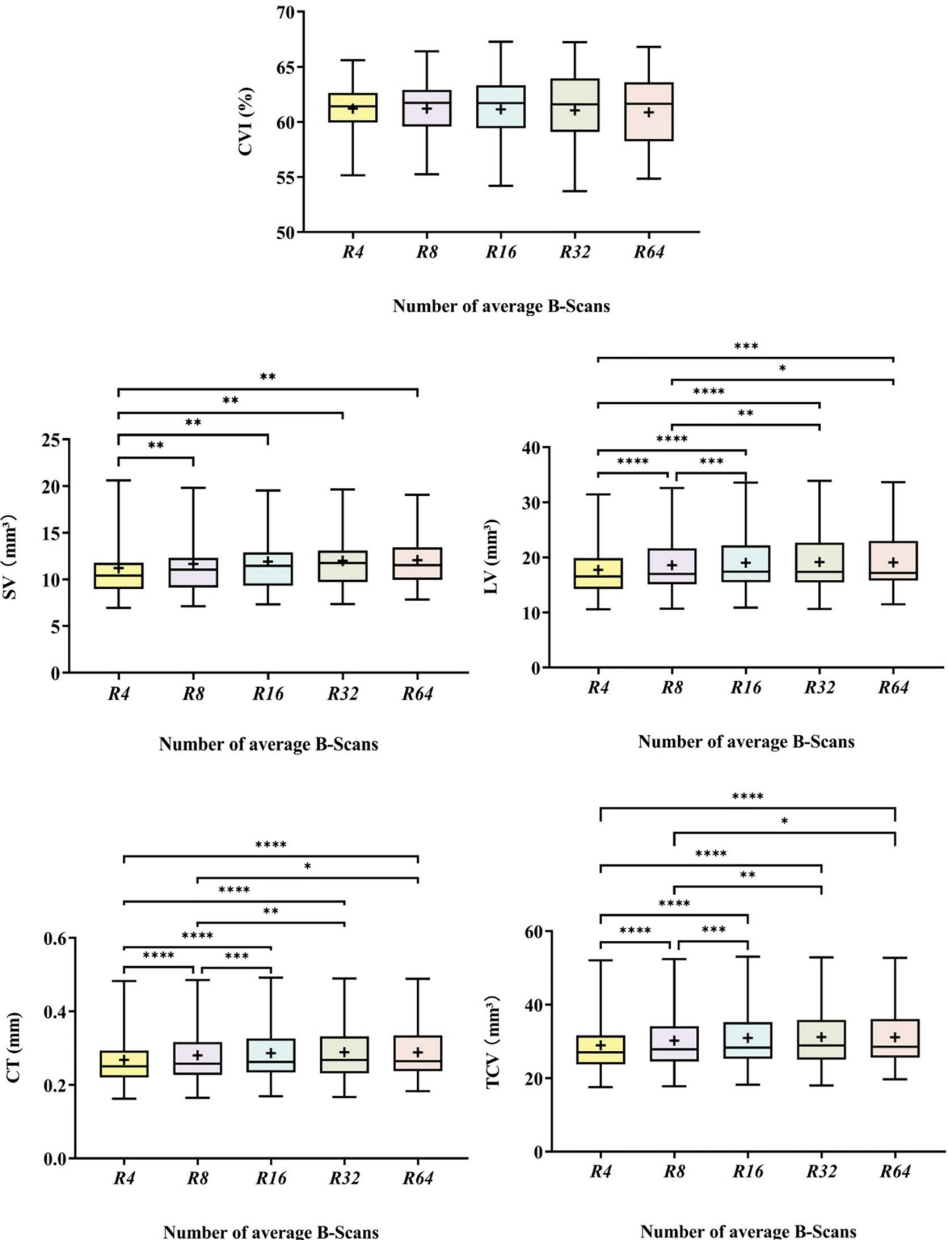
The impact of different B-scan averaging numbers (R4, R8, R16, R32, R64) in SS-OCT on choroidal parameters. The choroidal parameters include CVI, LV, SV, CT, and TCV. The box represents the interquartile range (IQR), encompassing the upper and lower quartiles; the horizontal line inside the box denotes the median. Whiskers indicate the minimum and maximum data points, and the “+” symbol represents the mean. “*, **, ***, ****” correspond to *P* < 0.05, *P* < 0.01, *P* < 0.001, and *P* < 0.0001, respectively, indicating statistical significance between groups

One-way repeated measure analysis of variance revealed significant differences in LV, SV, TCV and CT (all *P* < 0.05), while no significant difference was observed for CVI (*P* > 0.05) among different image averaging conditions (Fig. 4).

As for SV, a significant difference was observed between R4 and other averaging times. The SV average value was 11.21 mm³ at R4, and it stabilized within a range of 11.65 to 12.05 mm³ when the averaging times increased. With regard to LV, TCV and CT, a significant difference was observed between R4 and R8 compared to other averaging times. As the averaging times increased, the LV value rose from 17.73 mm³ at R4 to 18.57 mm³ at R8, then fluctuated between 19.01 and 19.15 mm³ with increases in averaging conditions, while the TCV value increased from 28.93 mm³ at R4 to 30.22 mm³ at R8, and fluctuated between 30.13 mm³ and 30.91 mm³. Similarly, CT increased from 268.14 μm at R4 to 280.06 μm at R8, and fluctuated between 286.45 μm and 288.48 μm as the averaging conditions increased.

## Discussion

To our knowledge, this is the first study to explore the impact of different B-scans averaging conditions on choroidal parameters like CVI using SS-OCT. In all B-scans averaging conditions, it demonstrates excellent repeatability and good consistency between two measurements within each group. We also find that the number of frames utilized for B-scan averaging does not affect CVI. However, for LV, TCV, and CT, a minimum of 16 frames is necessary to achieve optimal consistency, whereas SV requires at least 8 frames.

The results for CoR and ICC demonstrated the high precision, consistency, and reliability of the measurements across all B-scan averaging groups. CoR values were consistently low, with those for CVI consistently below 2%, volumetric parameters (LV, SV, and TCV) ranging from 0.435 to 1.188 mm³, and CT ranging from 0.010 to 0.011 mm. Similarly, the ICC values were all greater than 0.9, indicating excellent intra-observer repeatability. Bland-Altman analysis further supported these results, reinforcing the reliability and robustness of the measurements. The intra-observer repeatability of all choroidal parameters did not vary significantly across the B-scan averaging conditions (*P* > 0.05). However, the repeatability of CVI decreased with an increase in the number of averaging conditions, potentially due to increased subject fatigue and eye movement [25]. The above results show our choroidal quantization analysis method has good repeatability, which ensures the reliability of the results of the choroidal parameters.

The calculation of LV and SV relied on the recognition of luminal and stromal area in the OCT image. Under low averaging conditions, the OCT image exhibited a significant amount of speckle noise [26]. When the image was averaged only 4 times, parts of the luminal area were misidentified as stromal areas due to the influence of speckle noise. As the number of averaging conditions increased, the speckle noise was mitigated by smoothing filters, leading to more accurate identification of the vascular lumen area and a significant increase in LV. However, when the number of B-scans averaged reached 16, the improvement in signal to noise ratio (SNR) became limited, and further changes in LV were not significant. Shirasawa and Podkowinski et al. also found that averaging 16–20 B-scans significantly improved image quality metrics compared to a single non-averaged image, with further increases in the number of frames for B-scan averaging not significantly enhancing the image quality [27, 28].

As the number of averaging conditions increased, LV was anticipated to rise, which would normally reduce SV. However, contrary to expectations, SV increased. This unexpected increase may be due to the thickening of CT resulting from image averaging. Matsuo and Copete et al. reported that SS-OCT measures higher CT compared to SD-OCT, likely due to its better ability to delineate the choroidal-scleral interface [29–31]. In our study, lower B-scan averaging conditions introduced more speckle noise, making it challenging to distinguish between choroidal signals and noise around the choroidal-scleral interface. This noise could cause an upward shift in the identified choroidal-scleral interface, leading to a lower measured CT. When more averaged B-scan frames are used, the CT can be measured accurately by effectively reducing noise [32], which is the reason that CT and TCV increased significantly at R4 and R8. As the number of image averaging conditions exceeded 16, the visualization of the choroidal-scleral interface remains stable, leading to no significant changes in CT and TCV. Hoseini-Yazdi et al. found no significant change in CT under different averaging conditions [33]. We hypothesize that the discrepancy may be due to the difference in the imaging range. Their study utilized a 14 mm range, whereas our study focused on a 6 mm range in the ocular fovea and employed 3D data. Thus, it is necessary to pay attention to the image averaging protocol when we compare the above parameters in different literatures.

The values of CVI were not significantly different (*p* > 0.05) under different image averaging conditions, which may be related to the year-on-year growth of LV, SV and TCV. It was found that when the number of B-scan frames increased from R4 to R8, the LV grew by 0.047 and the TCV by 0.044, while the CVI, as a proportionality index reflecting choroidal structure, remained stable, demonstrating its robustness to noise and minor signal fluctuations. Although the averaging process of image superimposition reduced noise, it did not affect the overall structure of the choroid. This view is supported by a previous study by Agrawal and Rupesh et al. who reported that CVI results were in good agreement between SS-OCT and SD-OCT [34]. In addition, CVI has higher stability compared to other parameters and is less affected by multiple variables [35]. Therefore, CVI is insensitive to the image averaging protocol, suggesting it has better comparability across studies.

There are several limitations in our study. The findings of this study may not be applicable to older individuals with media opacity, reduced vision, or unstable fixation. Recent reports suggest that a greater number of B-scans are necessary to enhance the image quality of retinal OCT images in the presence of media opacity [28]. Additionally, the present study was limited by the small sample size, which may have reduced the statistical power to detect significant differences in choroidal thickness across various image averaging conditions.

In conclusion, this study provides evidence for the optimal number of B-scans needed for frame averaging to obtain repeatable and accurate measurements of choroidal parameters in healthy young eyes. Our results demonstrate that different choroidal parameters require specific minimum B-scan averaging levels to achieve measurement stability - with SV stabilizing at R8, while LV, TCV, and CT stabilize at R16. CVI showed no significant differences across averaging conditions (*P* > 0.05), confirming its relative stability. These findings have important clinical implications for standardizing choroidal imaging protocols, particularly when evaluating choroidal changes in various chorioretinal diseases such as pathological myopia, age-related macular degeneration, diabetic retinopathy, central serous chorioretinopathy, and glaucoma. While parameter-specific protocols might be justified in research settings, the practical limitations of extended scanning time and potential for increased patient fatigue and motion artifacts during image acquisition need to be considered. Our data show that 16-frame averaging (R16) represents a threshold at which all choroidal parameters (LV, SV, TCV, CT, and CVI) demonstrate statistical stability. Higher averaging levels (R32, R64) did not yield statistically significant differences in measurement values while extending scan duration. Conversely, lower averaging levels (R4, R8) were insufficient for parameters such as LV, TCV, and CT. The stability of CVI across different averaging conditions suggests this parameter may be particularly suitable when comparing measurements from different imaging protocols. These observations provide an evidence base that can guide the selection of appropriate B-scan averaging protocols and choroidal quantitative parameters in future research, potentially improving the reliability and comparability of choroidal measurements across different studies.

## Data Availability

The datasets used and/or analysed during the current study are available from the corresponding author on reasonable request.

## Abbreviations

OCT: Optical coherence tomography
SS-OCT: Swept-source optical coherence tomography
SD-OCT: Spectral-domain OCT
CVI: Choroidal vascularity index
CT: Choroidal thickness
LV: Luminal volume
SV: Stromal volume
TCV: Total choroidal volume
LA: Luminal area
SA: Stromal area
TCA: Total choroidal area
CSI: Choroidal stromal index
BCVA: Best-corrected visual acuity
RPE: Retinal pigment epithelium
ICC: Intraclass correlation coefficients
CoR: Coefficient of repeatability
SD: Standard deviation
CI: Confidence interval
LoA: Limits of agreement
ANOVA: Analysis of variance
SNR: Signal to noise ratio
IQR: Interquartile range
R4: 4 repeated B-scans for averaging
R8: 8 repeated B-scans for averaging
R16: 16 repeated B-scans for averaging
R32: 32 repeated B-scans for averaging
R64: 64 repeated B-scans for averaging
